# Supplementary material for: Emergent competition shapes top-down versus bottom-up control in multi-trophic ecosystems
Source: PLoS Comput Biol. 2024 Feb 8;20(2):e1011675. doi: 10.1371/journal.pcbi.1011675 (PMC10852287; doi:10.1371/journal.pcbi.1011675)
Supplement: S1 Text — In this section, we analyze the special case below where there is only 1 species with intra-species competition on each level. (PDF) [file pcbi.1011675.s001.pdf]

# Emergent competition shapes top-down versus bottom-up control in multi-trophic ecosystems

Zhijie Feng[1], Robert Marsland, III[1¤], Jason W. Rocks[1], Pankaj Mehta [1,2,3*],

**1** Department of Physics, Boston University, Boston, MA 02215, USA
**2** Biological Design Center, Boston University, Boston, MA 02215, USA
**3** Faculty of Computing and Data Science, Boston University, Boston, MA 02215, USA

¤Current Address: Pontifical University of the Holy Cross, Rome, Italy
* pankajm@bu.edu

## Contents

**1 Top-down and bottom-up control in toy model** **1**

**2 Additional Figures** **3**
2.1 SI Figure 1 . . . . . . . . . . . . . . . . . . . . . . . . . . . . . . . . . 3
2.2 SI Figure 2 . . . . . . . . . . . . . . . . . . . . . . . . . . . . . . . . . 4
2.3 SI Figure 3 . . . . . . . . . . . . . . . . . . . . . . . . . . . . . . . . . 5
2.4 SI Figure 4 . . . . . . . . . . . . . . . . . . . . . . . . . . . . . . . . . 6
2.5 SI Figure 5 . . . . . . . . . . . . . . . . . . . . . . . . . . . . . . . . . 7

## 1 Top-down and bottom-up control in toy model

In this section, we analyze the special case below where there is only 1 species with intra-species competition on each level. Similar analysis has also been done in [9].

$$
\begin{aligned}
\frac{dX_1}{dt} &= X_1(-u_1 - D_X X_1 + \eta_X d_{11} N_1),\\
\frac{dN_1}{dt} &= N_1(-m_1 - D_N N_1 + \eta_N c_{11} R_1 - d_{11} X_1),\\
\frac{dR_1}{dt} &= R_1(k_1 - D_R R_1 - c_{11} N_1).
\end{aligned}
\tag{S1}
$$

The conditions for steady states are

$$
\begin{aligned}
0 &= X_1(-u_1 - D_X X_1 + \eta_X d_{11} N_1),\\
0 &= N_1(-m_1 - D_N N_1 + \eta_N c_{11} R_1 - d_{11} X_1),\\
0 &= R_1(k_1 - D_R R_1 - c_{11} N_1).
\end{aligned}
\tag{S2}
$$

To see the top-down and bottom-up effect, we can obtain the stable steady state in the region where everything survives (i.e. $R_1 > 0, N_1 > 0, X_1 > 0$) :

$$
\begin{aligned}
X_1 &= -\frac{c_{11}^2 \eta_N u_1 - c_{11} d_{11} \eta_N \eta_X k_1 + d_{11} D_R \eta_X m_1 + D_R D_N u_1}{c_{11}^2 D_X \eta_N + d_{11}^2 D_R \eta_X + D_R D_N D_X},\\
R_1 &= -\frac{c_{11} d_{11} u_1 - c_{11} D_X m_1 - d_{11}^2 \eta_X k_1 - D_N D_X k_1}{c_{11}^2 D_X \eta_N + d_{11}^2 D_R \eta_X + D_R D_N D_X},\\
N_1 &= -\frac{-c_{11} D_X \eta_N k_1 - d_{11} D_R u_1 + D_R D_X m_1}{c_{11}^2 D_X \eta_N + d_{11}^2 D_R \eta_X + D_R D_N D_X},
\end{aligned}
\tag{S3}
$$

and take the derivative with respect to $k_1$ and $u_1$ to obtain an order parameter

$$\frac{\frac{dN_1}{du_1}}{\frac{dN_1}{dk_1}+\frac{dN_1}{du_1}} = \frac{d_{11}D_R}{d_{11}D_R + c_{11}D_X\eta_N}. \tag{S4}$$

We can see from here that top-down control exceeds bottom-up control when $d_{11}D_R > c_{11}D_X\eta_N$, and vice versa. For instance, in Fig. S1(a), if species coexists (approximately $u_1 < 3, k_1 > 4$), we can see there is much more bottom-up control, as $\frac{|\frac{dN_1}{du_1}|}{|\frac{dN_1}{dk_1}|+|\frac{dN_1}{du_1}|} = \frac{4*1}{4*1+4*5*0.9} \approx 0.18 < 0.5$. In contrast, there is much more top-down control in Fig. S1(b), as $\frac{|\frac{dN_1}{du_1}|}{|\frac{dN_1}{dk_1}|+|\frac{dN_1}{du_1}|} = \frac{9*3}{9*3+4*3*0.9} \approx 0.71 > 0.5$. It is worth noting that, unlike the multi-species case in the main text, the derivatives are independent of $k_1, u_1$ in this coexistence region.

If we continuously change the parameters, we can also observe a bifurcation to the region where only herbivore and plant survive (i.e. $R_1 > 0, N_1 > 0, X_1 = 0$):

$$\begin{aligned} X_1 &= 0, \\ N_1 &= \frac{c_{11}\eta_N k_1 - D_R m_1}{D_N D_R + c_{11}^2\eta_N}, \\ R_1 &= \frac{D_N k_1 + c_{11} m_1}{D_N D_R + c_{11}^2\eta_N}. \end{aligned} \tag{S5}$$

Here there is no top-down control as $N_1$ is independent of $u_1$, trivially due to the absence of top level. In Fig. S1, we can observe this when approximately when $u_1 > 4, k_1 < 2$. With more extreme choice of parameters, there is also region where only plant survives ($R_1 = k_1/D_R, N_1 = X_1 = 0$), or nothing survives,( $R_1 = N_1 = X_1 = 0$).

## 2 Additional Figures

### 2.1 SI Figure 1

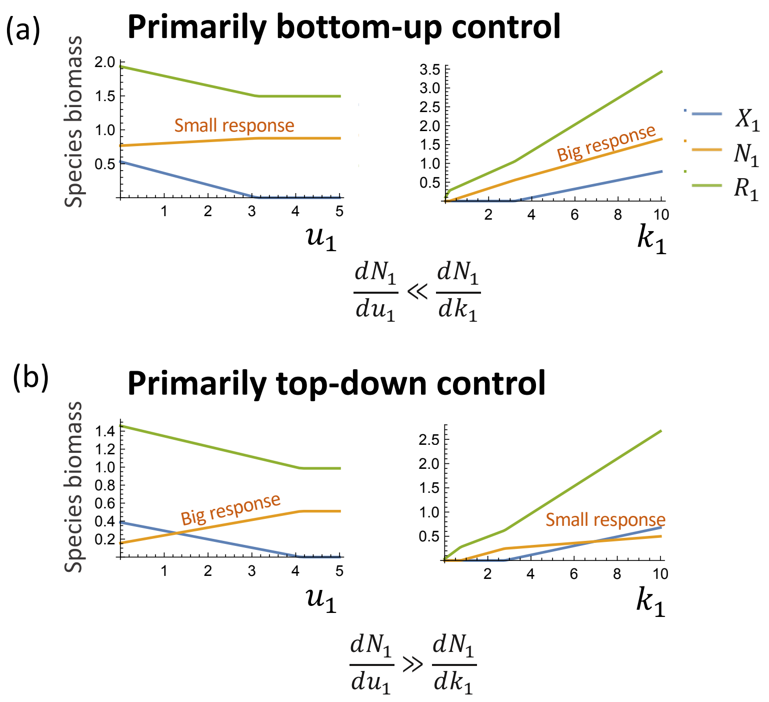


**Fig S1. Demonstration of bottom-up control and top-down control analogous to Fig. 3 for special case with single species on each level. (a)** Exact biomass at each trophic level as a function of $u_1$ with $k_1 = 5, m_1 = 1, \eta_N = \eta_X = 0.9, D_R = 1, D_X = 5, D_C = 5.c_{11} = 4, d_{11} = 4$ (left), and as a function of $k_1$ with $u_1 = 2$ (right). **(b)** Same as (a) except $D_R = 3, D_X = 3, d_{11} = 9$

## 2.2 SI Figure 2

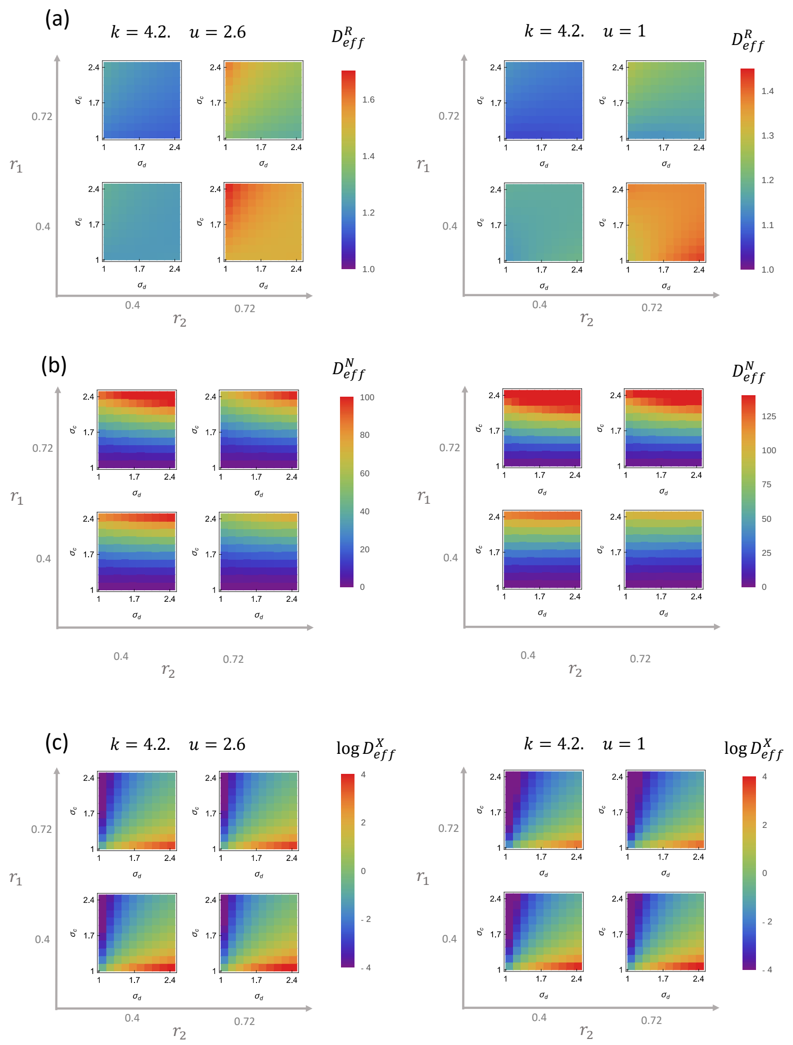


**Fig S2. Plot of effective competition coefficients.** **(a)** $D^R_{eff}$, **(b)** $D^N_{eff}$ and **(c)** $D^X_{eff}$ under the same conditions as Fig. 4.

## 2.3 SI Figure 3

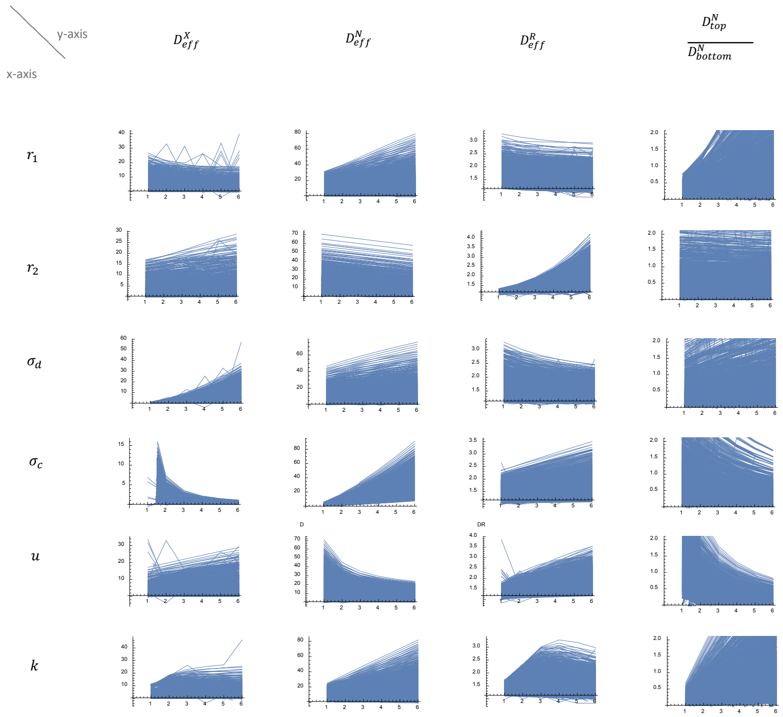


**Fig S3. Plots of effective quantities versus model parameters**. Plot of $D^X_{eff}$, $D^N_{eff}$, $D^R_{eff}$, and $D^N_{top}/(D^N_{top} + D^N_{bottom})$ versus $r_1 \in [0.4, 1.2]$, $r_2 \in [0.4, 1.2]$, $\sigma_d \in [1, 2.5]$, $\sigma_c$, $u \in [1, 5]$, and $k \in [1, 5]$, obtained from evaluating the cavity solutions with all 6 parameters varied simultaneously, each with 6 possible values in the range. Each trajectory correspond to varying the parameter on it's x-axis, while fixing the other 5 parameters. These plots arranged in table directly maps to the result in Table 1 .

## 2.4 SI Figure 4

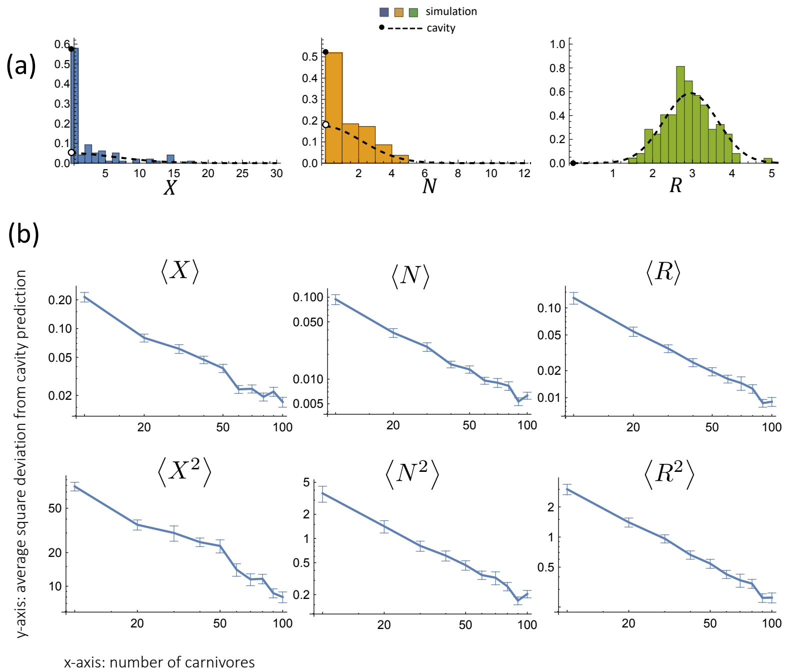


**Fig S4. System size affects cavity solution convergence.**(a)Histograms of the steady state reached by dynamics of a system with $M_X = 50$ species of carnivores, $M_N = 56$ herbivores and $M_R = 62$ plants, with $k = 4, m = 1, u = 1, \sigma_c = \sigma_d = 0.5, \mu_c = \mu_d = 1, \eta_X = 1, \eta_N = 1, \sigma_k = \sigma_m = \sigma_u = 0.1$, and the distribution predicted by our cavity solution. Note that a black dot correspond the finite extinction probability (instead of probability density) predicted by cavity solution, while a black dash correspond to the probability density(b) The average square deviation of the single system statistics from cavity solution as a function of $M_X$ while keeping fixed ratios $r_1 = r_2 = 0.9$, averaged from 200 sample systems.

### 2.5 SI Figure 5

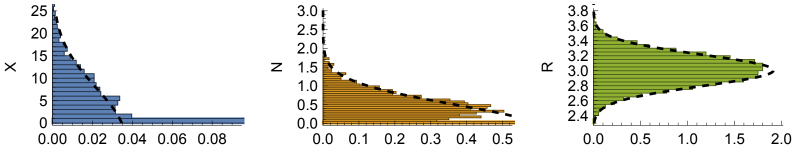


**Fig S5. Typical distributions of biomass with uniform distributions of consumer preference matrix elements also agrees well with cavity method.** Solutions shown with parameters $k = 4$, $u = 1$, $\sigma_c = 0.5$, $\mu_c = 5$, $\sigma_d = 0.5$, $\mu_d = 5$, $r_1 = 1$, $r_2 = 1$ and sampled from 200 systems with 30 species in each level.
