# Supplementary figures and images for: Emergent competition shapes top-down versus bottom-up control in multi-trophic ecosystems
Source: PLoS Comput Biol. 2024 Feb 8;20(2):e1011675. doi: 10.1371/journal.pcbi.1011675 (PMC10852287; doi:10.1371/journal.pcbi.1011675)

(a) **Primarily bottom-up control**

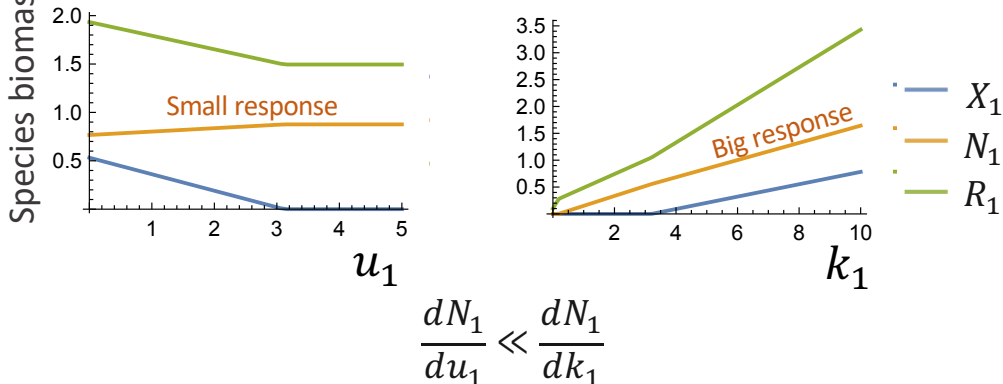

(b) **Primarily top-down control**

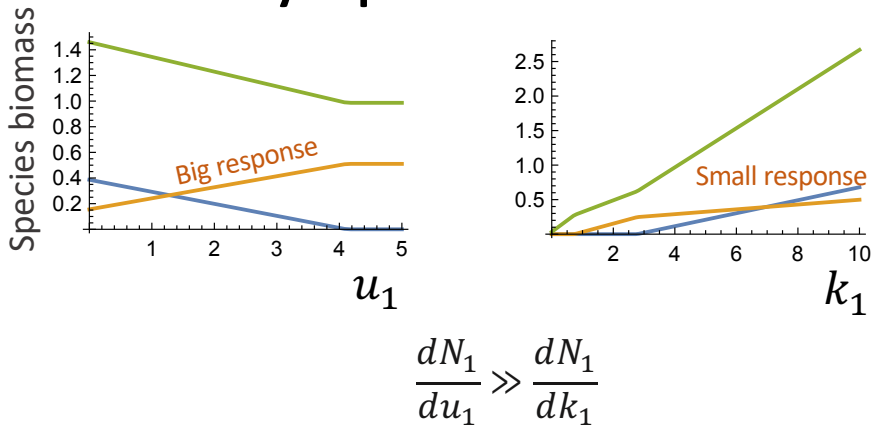

Supplement: S1 Fig — (a) Exact biomass at each trophic level as a function of u1 with k1 = 5, m1 = 1, ηN = ηX = 0.9, DR = 1, DX = 5, DC = 5.c11 = 4, d11 = 4 (left), and as a function of k1 with u1 = 2 (right). (b) Same as (a) except DR = 3, DX = 3, d11 = 9. (PDF) [file pcbi.1011675.s002.pdf]

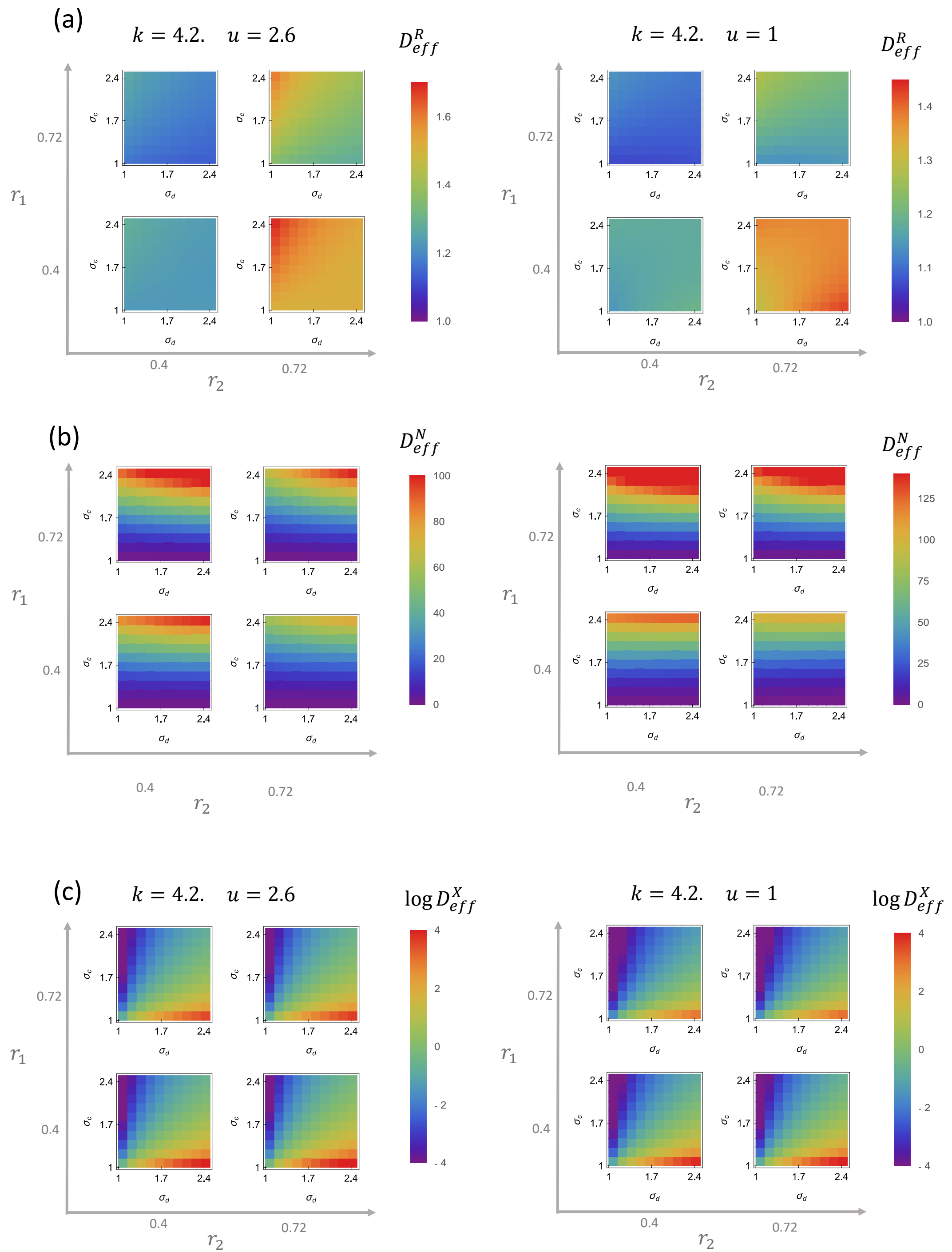

Supplement: S2 Fig — (PDF) [file pcbi.1011675.s003.pdf]

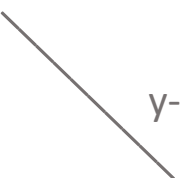
  
 x-axis
   
 y-axis

$D_{eff}^X$

$D_{eff}^N$

$D_{eff}^R$

$D_{top}^N$ 
  
 $\overline{D_{bottom}^N}$

$r_1$

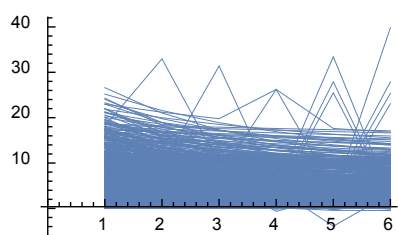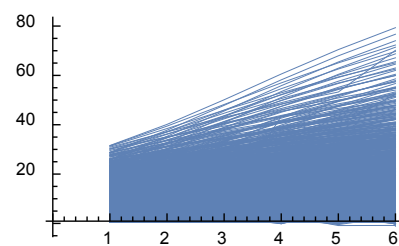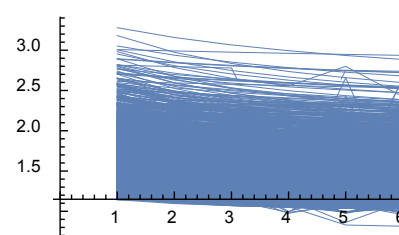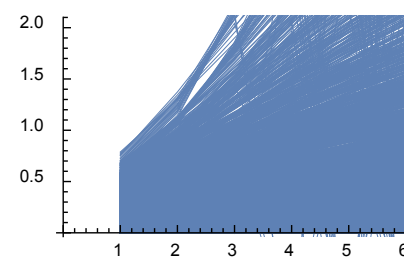

$r_2$

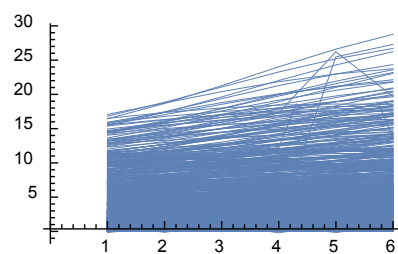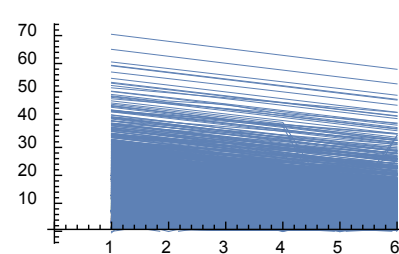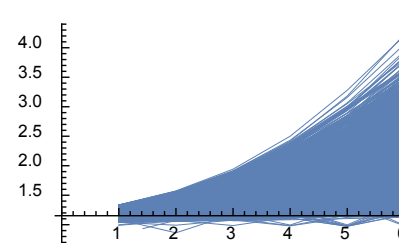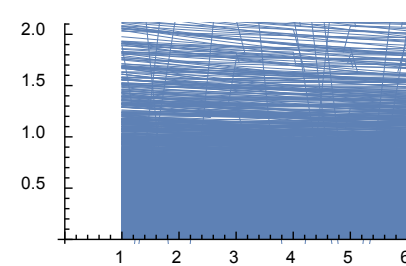

$\sigma_d$

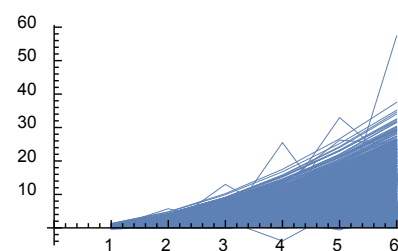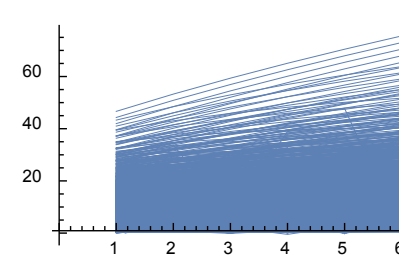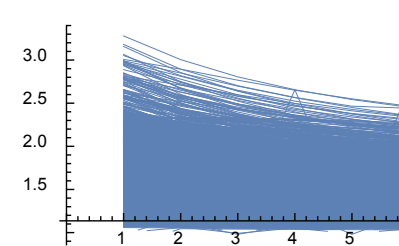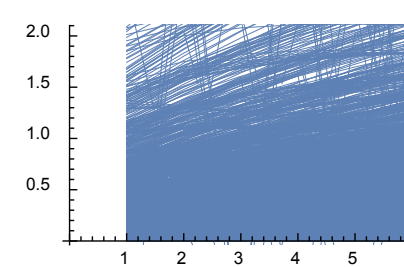

$\sigma_c$

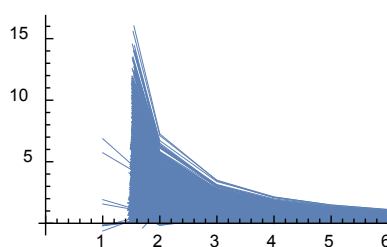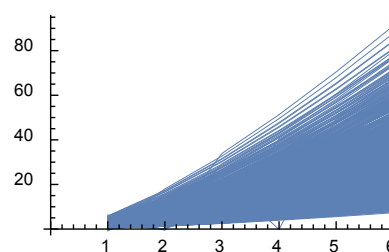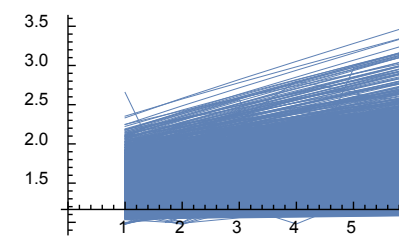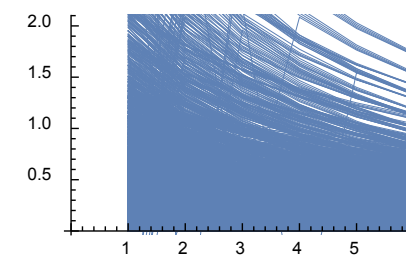

$u$

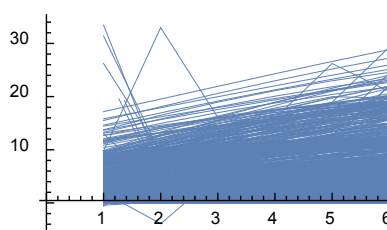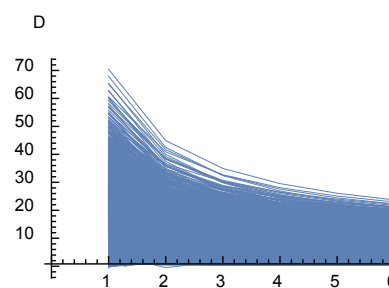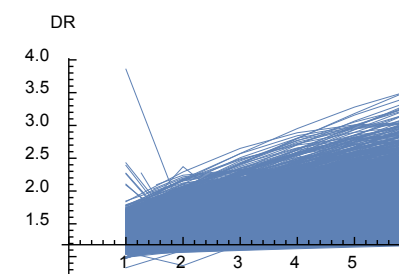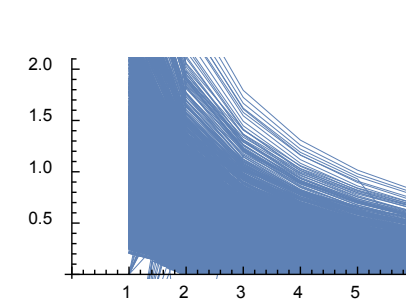

$k$

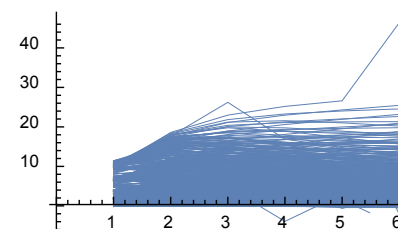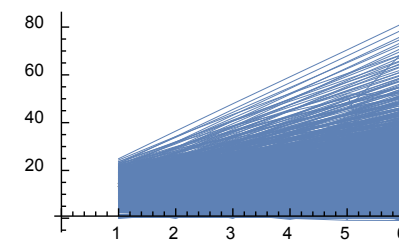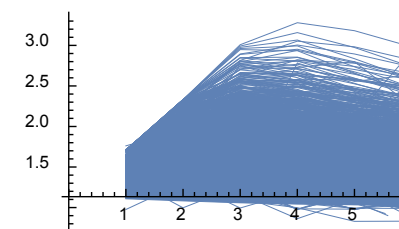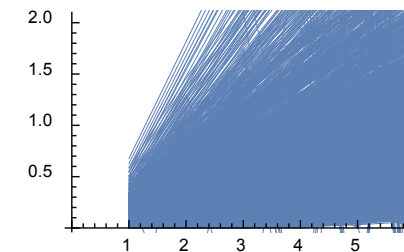

Supplement: S3 Fig — Plot of DeffX, DeffN, DeffR, and DtopN/(DtopN+DbottomN) versus r1 ∈ [0.4, 1.2], r2 ∈ [0.4, 1.2], σd ∈ [1, 2.5], σc, u ∈ [1, 5], and k ∈ [1, 5], obtained from evaluating the cavity solutions with all 6 parameters varied simultaneously, each with 6 possible values in the range. Each trajectory correspond to varying the parameter on it’s x-axis, while fixing the other 5 parameters. These plots arranged in table directly maps to the result in Table 1. (PDF) [file pcbi.1011675.s004.pdf]

(a)

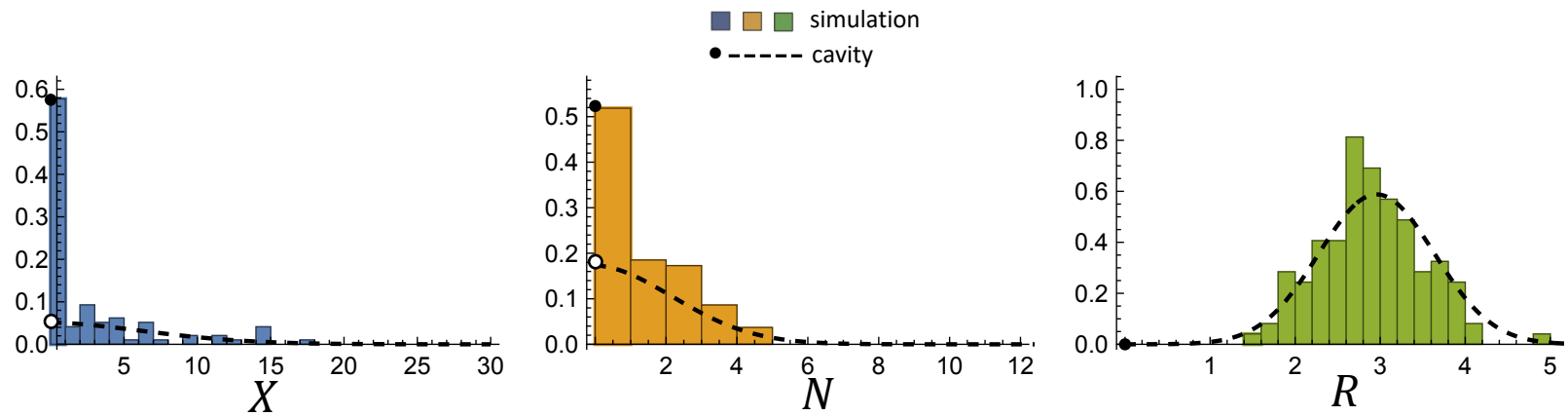

(b)

y-axis: average square deviation from cavity prediction

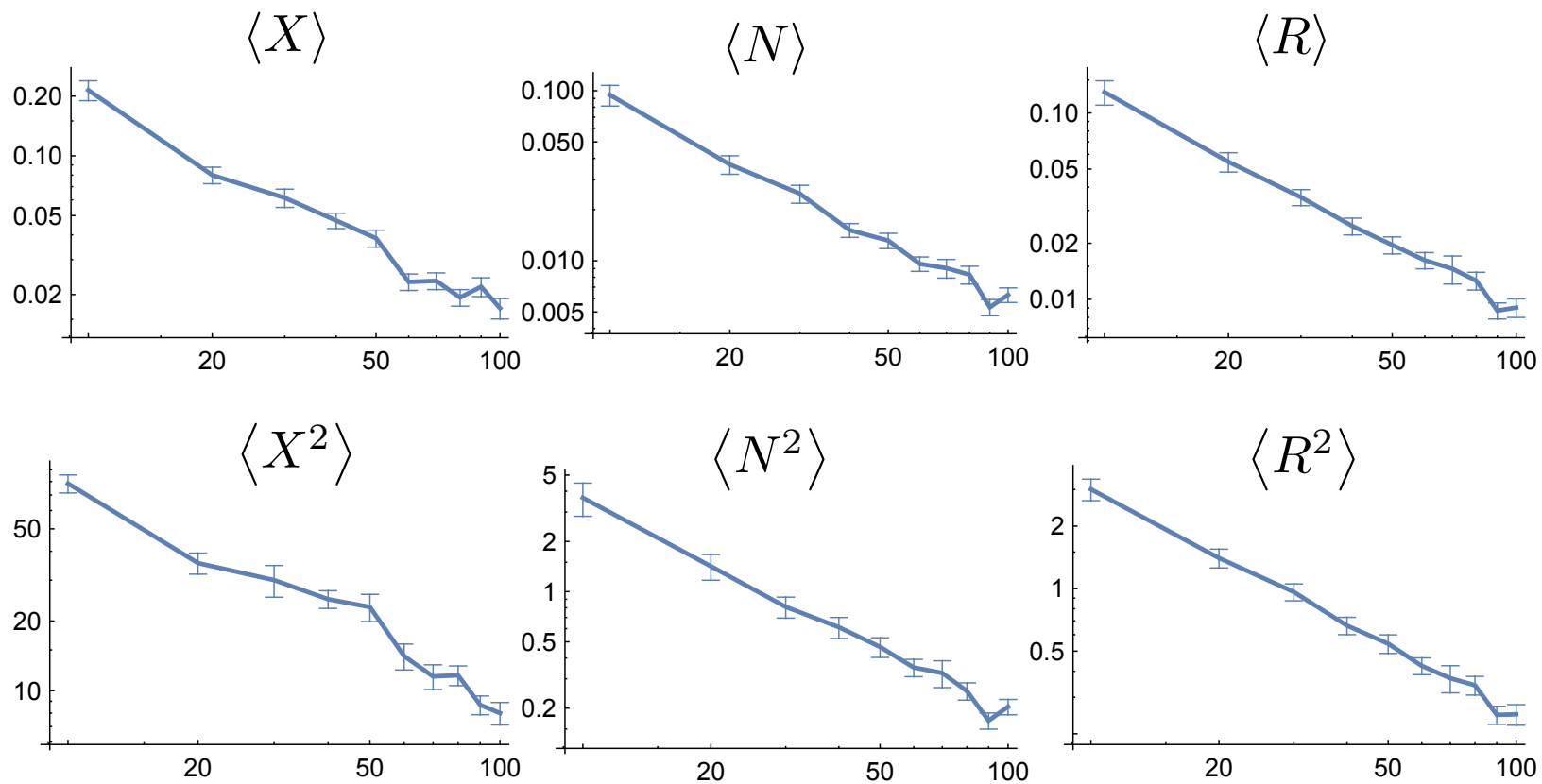

x-axis: number of carnivores

Supplement: S4 Fig — (a)Histograms of the steady state reached by dynamics of a system with MX = 50 species of carnivores, MN = 56 herbivores and MR = 62 plants, with k = 4, m = 1, u = 1, σc = σd = 0.5, μc = μd = 1, ηX = 1, ηN = 1, σk = σm = σu = 0.1, and the distribution predicted by our cavity solution. Note that a black dot correspond the finite extinction probability (instead of probability density) predicted by cavity solution, while a black dash correspond to the probability density(b) The average square deviation of the single system statistics from cavity solution as a function of MX while keeping fixed ratios r1 = r2 = 0.9, averaged from 200 sample systems. (PDF) [file pcbi.1011675.s005.pdf]

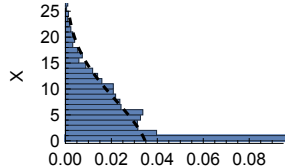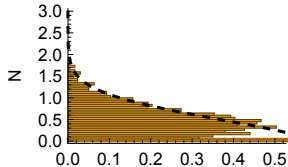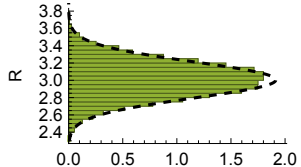

Supplement: S5 Fig — Solutions shown with parameters k = 4, u = 1, σc = 0.5, μc = 5, σd = 0.5, μd = 5, r1 = 1, r2 = 1 and sampled from 200 systems with 30 species in each level. (PDF) [file pcbi.1011675.s006.pdf]
