# Supplementary material for: Emergent competition shapes top-down versus bottom-up control in multi-trophic ecosystems
Source: PLoS Comput Biol. 2024 Feb 8;20(2):e1011675. doi: 10.1371/journal.pcbi.1011675 (PMC10852287; doi:10.1371/journal.pcbi.1011675)
Supplement: Attachment — Submitted filename: review-feng2023.pdf [file pcbi.1011675.s007.pdf]

Review of "Emergent competition shapes the ecological properties of multi-trophic ecosystems"

The authors introduce a consumer resource model consisting of three trophic levels, each populated by large random communities, and obtain an analytical description of different aspects of the model ecosystem by means of the cavity method, supported by numerical simulations. The authors characterize the effective competition within each trophic level which emerges from the interaction among trophic levels and provide insights on which kind of control (top-down or bottom-up) is relevant in a given community based on coarse properties of the ecosystem.

The results presented are well-supported, clear and of ecological significance. The paper is extremely well written, effective in conveying the results and technically sound. Finally, the exhaustive Appendix and the care devoted to technical aspects make it a valuable methodological resource, bringing additional value beyond the results presented.

The manuscript is of certain interest for the readers of PLOS Computational Biology and I strongly recommend its publication. I only have one comment, which is possibly more about form than substance, plus some minor comments of various nature. I believe it would be beneficial to the readers if the authors address them prior to publication.

Comment:

I believe that the emphasis (e.g. in title, abstract and conclusions) on "emergent competition" arising in a trophic level as feedback from interactions with other trophic levels is a bit misplaced. This is not a new concept and the authors could be more cautious in the wording in order to not let it appear as such (as an example, the sentence in the abstract "We find that intra-trophic diversity gives rise to “emergent competition” between species within a trophic level due to feedbacks mediated by other trophic levels" can easily convey the message that this is an original result of this paper). Indeed this has been known and formalized at least since Ref. [MacArthur, Robert. "Species packing and competitive equilibrium for many species." *Theoretical population biology* 1.1 (1970): 1-11.] (which should be included), where MacArthur derives competitive GLV from a consumer resource model. The essence of emergent competition is already there. The fact that more than two trophic levels are considered, and that cavity method is used to obtain an effective analytical description of typical communities in the case of random interactions (admittedly allowing the author to build really clear and fresh intuition on various details of the emergent competition) are not necessary ingredients for that purpose.

On the other hand, for example, the insights on the type of control in the ecosystems, possible thanks to the cavity-inspired order parameters, are extremely interesting and worth to be given even more relevance.

I believe that the paper would benefit, in clarity and effectiveness in conveying the novelty introduced, from a removal or small rewriting of sentences related to the argument above, and, maybe, a modification of the title.

Minor comments:

- In Fig. 1 (b) the "Herbivore" box should be in yellow and not orange to avoid confusion with the environment color code. And, maybe, the gray arrows here and in figure (a) could be in yellow as well.
- The author could probably stress even more than they already do that Eq. (6) is valid only at steady state and cannot be interpreted as an effective dynamical equation for the system away from the steady state. Of course the author are well aware of this and therefore write

$$0 = \frac{dX}{dt} = \dots$$

etc... but maybe an additional sentence to highlight this fact can go a long way to avoid possible misinterpretations.

- In caption of Table I maybe it can be specified that Figs. 5 and 6 are in the Appendix.
- Fig. 4 is mentioned in the main text before Fig.3. They should be switched.
- Typo in Section IV C. In the sentence "This suggests that the order parameter $D_{eff}^{N,top}/D_{eff}^{N,bottom}1$ ... " contains a "1" which should be removed: $D_{eff}^{N,top}/D_{eff}^{N,bottom}1 \to D_{eff}^{N,top}/D_{eff}^{N,bottom}$.
- In the caption of Table II the author should specify that Figures 5 and 6 are in the Appendix.
- In the Appendix, Ref. [26] is mentioned in two places and in both cases square brackets are missing: Ref. 26 $\to$ Ref. [26].
- In the Appendix, first sentence of section 3 there are missing parenthesis: Eqs. A17-A19 $\to$ Eqs. (A17)-(A19).
- The caption of Fig. 6 may need improvements. Let us consider the plot relative to D_{eff}^{X} and r_1. If I understand correctly, a "trajectory" here refers to a choice of the other 5 parameters (and, possibly, there is more than one trajectory sampled for each combination) for increasing value of r_1, from its 1st to its 6th value. Either if this is the case, or something else is instead plotted here, it will be helpful for the readers to have a clearer and more explicit description of the plots.
- In the caption of Fig. 6 a period is missing at the end after "Table I".
- References [24] and [30] are the same. (Moreover, typesetting of Ref. [30] is wrong).
