# Supplementary material for: Emergent competition shapes top-down versus bottom-up control in multi-trophic ecosystems
Source: PLoS Comput Biol. 2024 Feb 8;20(2):e1011675. doi: 10.1371/journal.pcbi.1011675 (PMC10852287; doi:10.1371/journal.pcbi.1011675)
Supplement: Attachment — Submitted filename: review_response.pdf [file pcbi.1011675.s008.pdf]

Dear Jacopo Grilli, Natalia Komarova,

Please find enclosed the revised version of our previous submission entitled "Emergent competition shapes the ecological properties of multi-trophic ecosystems" with manuscript number PCOMPBIOL-D-23-00463. We would like to thank you and the reviewers for the valuable comments which help improving the quality of our manuscript. In this revision, we have carefully addressed the reviewers' comments.

We have made extensive revisions, including: generalizing our calculations to account for the possibility of inefficient energy transfers between trophic layers (through the introduction of the parameters η_X and η_N), making new plots that treat regions of top-down and bottom-up control in phase diagram more symmetrically, performing simulations for finite system sizes, and extensive minor clarifications and revisions.

A summary of main modifications and a detailed point-by-point response to the comments from reviewers are given below.

Sincerely,

Zhijie Feng, Robert Marsland III, Jason W. Rocks, and Pankaj Mehta

Note: For clarity, each comment is displayed in a blue box, and additions to the manuscript are in gray boxes.

Authors' Response to Reviewer 1

Comment 1

The general reader would benefit from some simplified examples. For example, the coupling between species at the central trophic level due to a shared resource or a shared predator could be explained using a model with only three species, in each case. Even more important would be a simple example of a situation leading to bottom up control vs. one leading to top down control. While ecologists may already understand these ideas, they are likely to be unfamiliar to many readers. Simple "toy models" that capture these behaviors would therefore be a big help. I leave it up to the authors whether some of these examples can be relegated to the supplement.

Response:

We thank the reviewer for this comment. We agree that a simplified example for building intuition is useful. We have added a section to the Appendix with a single species at each trophic layer.

We have added new section to Appendix with a simplified example illustrating bottom up and top down control.

Comment 2

There are a great number of variables, so simplification of the notation would be helpful. For example, in Eq. 1 it would be easier to parse of Q was replaced by P, beta by alpha, and j by i. I don't see any problem with using the same variable name in the summations, and it would be easier to understand the equations. (In fact, the authors have made at least one mistake by having too many variables - in the last box of Fig. 1e the Q should be a P. Above Eq. 2, the two appearances of $d_i a$ should be $d_\alpha i$).

Response:

We agree that simplifying the equations is helpful. To this end, we have simplified the cavity equations by substituting all the denominators terms with effective terms $D_{eff}^{(\cdot)}$. We also fixed several typos related to the indices.

Typo of indexs has been fixed in Equation 2 and Figure 1. Simplification to Equation A10-A19, and A24-A27.

Comment 3

The histograms in Fig. 1c appear to be the combined results of many independent simulations (and this number should be stated in the caption). However, a given ecosystem only has one set of species. What does that distribution look like for one realization of parameters? It would be helpful to show such individual distributions - ideally along with some measure of agreement between individual distributions and the expected average distribution - as a function of the total number of species. Also, in Fig. 1c, there is a difference between the simulation results and the cavity results for X and N at the lowest bar in the histogram. Given the essentially perfect agreement for the rest of the distribution, some discussion of this deviation is called for.

Response:

We have added a figure in the appendix (Figure 8) to show individual distribution, and the square deviation from prediction as a function of number of species. The lowest bar corresponds to the extinct species. As can be seen from the figure, there is actually very little deviation from the analytic results. There are large peaks at 0, not shown to full scale in Figure 1 for visual clarity. We have added a note to the figure caption to indicate this. We have also added a paragraph to the main text indicating that finite size effects are small.

Caption for Figure 1: "For visual clarity, they are not shown to full scale, but also agree with the cavity predictions (see appendix Fig. 1)."
We also show the agreement of this peak in full scale in the new figure, Figure 8(a).

In main text: "Our calculations are exact in the thermodynamic limit where there are infinite number of species in the regional pool at each trophic level. To understand the finite size corrections, we performed numerical simulations for ecosystems of different sizes. As can be seen in appendix Fig. 8, our analytic predictions agree well with numerics even for modest size ecosystems with of order 20 species."

Comment 4

There is a great deal of discussion in the ecology literature about rank-abundance curves, so it would be appropriate to discuss how the distributions of species abundances in the current model compare to actual rank-abundance curves.

Response:

This is an interesting point. However, to despite our efforts, we could not find any quantitative rank-abundance curves for multi trophic ecosystems. However, we have added a sentence to the discussion that this might be an interesting direction.

> It may be also interesting to fit the model to actual ecosystems, for instance by making use of rank-abundance curves data for real systems to fix the biomass distributions of the model.

Comment 5

There are a few misspellings and missing words, so another careful reading of the manuscript would be helpful.

Response:

We have tried our best to fix typos and misspellings.

Authors' Response to Reviewer 2

Comment 1

I believe that the emphasis (e.g. in title, abstract and conclusions) on "emergent competition" arising in a trophic level as feedback from interactions with other trophic levels is a bit misplaced. This is not a new concept and the authors could be more cautious in the wording in order to not let it appear as such (as an example, the sentence in the abstract "We find that intra-trophic diversity gives rise to “emergent competition” between species within a trophic level due to feedbacks mediated by other trophic levels" can easily convey the message that this is an original result of this paper). Indeed this has been known and formalized at least since Ref. [MacArthur, Robert. "Species packing and competitive equilibrium for many species." Theoretical population biology 1.1 (1970): 1- 11.] (which should be included), where MacArthur derives competitive GLV from a consumer resource model. The essence of emergent competition is already there. The fact that more than two trophic levels are considered, and that cavity method is used to obtain an effective analytical description of typical communities in the case of random interactions (admittedly allowing the author to build really clear and fresh intuition on various details of the emergent competition) are not necessary ingredients for that purpose. On the other hand, for example, the insights on the type of control in the ecosystems, possible thanks to the cavity-inspired order parameters, are extremely interesting and worth to be given even more relevance. I believe that the paper would benefit, in clarity and effectiveness in conveying the novelty introduced, from a removal or small rewriting of sentences related to the argument above, and, maybe, a modification of the title.

Response:

We agree that the concept that effective competition emerging between consumers due to diversity is one of the most important results of theoretical ecological works of 1960s and 1970s (especially the pioneering works of MacArthur and Levins). We are big fans of these papers and they served as a direct inspiration for this work. The original title and abstract was meant to emphasize that we could absorb the effect of diversity in effective TAP terms.

We have added the reference suggested, adjusted wording and add sentences. In the aforementioned reference, the method was to substitute the steady state of resources into the equation of consumers, which produce an effective GLV model. While the ecological concept is the same, our results are qualitatively different in a few ways. The most important of these is that the competition intensities in our work are emergent in the sense that they only depend on community-level

(statistical) properties of the system, and they comes from coarse-graining of large systems. Namely, we have derived mean-field equations of a single effective species at each trophic layer rather than a GLV for a collection of species.

That being said, we agree with the reviewer that we should emphasize the novel results and features of our cavity calculation. For this reason, we have both changed the title and the abstract to emphasize .

Our new title is now: "Emergent competition shapes top-down versus bottom-up control in multi-trophic ecosystems".

Our abstract now reads:
"Ecosystems are commonly organized into trophic levels – organisms that occupy the same level in a food chain (e.g., plants, herbivores, carnivores). A fundamental question in theoretical ecology is how the interplay between trophic structure, diversity, and competition shapes the properties of ecosystems. To address this problem, we analyze a generalized Consumer Resource Model with three trophic levels using the zero-temperature cavity method and numerical simulations. **We derive the corresponding mean-field cavity equations and show that intra-trophic diversity gives rise to an effective "emergent competition" term between species within a trophic level due to feedbacks mediated by other trophic levels.** This emergent competition gives rise to a crossover from a regime of top-down control (populations are limited by predators) to a regime of bottom-up control (populations are limited by primary producers) and is captured by a simple order parameter related to the ratio of surviving species in different trophic levels. We show that our theoretical results agree with empirical observations, suggesting that the theoretical approach outlined here can be used to understand complex ecosystems with multiple trophic levels. "

Comment 2

In Fig. 1 (b) the "Herbivore" box should be in yellow and not orange to avoid confusion with the environment color code. And, maybe, the gray arrows here and in figure (a) could be in yellow as well.

Response:

Thank you for the suggestion. We have changed the color scheme so that arrows for dissipation or energy inflow always has the same color as trophic level.

Comment 3

The author could probably stress even more than they already do that Eq. (6) is valid only at steady state and cannot be interpreted as an effective dynamical equation for the system away from the steady state. Of course the author are well aware of this and therefore write $0 = \frac{dX}{dt} = \ldots$ etc... but maybe an additional sentence to highlight this fact can go a long way to avoid possible misinterpretations.

Response:

We have added the following sentence to make this clear.

> "We emphasize that these equations are only valid at steady-state and capturing the actual coarse-grained dynamics would involve solving for the full dynamical mean-field theory equations."

Comment 4

In caption of Table I maybe it can be specified that Figs. 5 and 6 are in the Appendix.

Response:

Thank you for this suggestion. We added the sentence in the caption.

Comment 5

Fig. 4 is mentioned in the main text before Fig.3. They should be switched.

Response:

One of the references to figure 4 in section IV B was actually a typo and we have corrected this in the new version of the manuscript.

Comment 6

Typo in Section IV C. In the sentence "This suggests that the order parameter $D_{eff}^{N,top}/D_{eff}^{N,bottom}$ contains a "1" which should be removed.

Response:

Thank your for your careful reading of the manuscript. We have removed the "1".

Comment 7

In the caption of Table II the author should specify that Figures 5 and 6 are in the Appendix.

Response:

We have added the sentence in the caption of Table II

Comment 8

In the Appendix, Ref. [26] is mentioned in two places and in both cases square brackets are missing: Ref. 26 → Ref. [26].

Response:

We have fixed the missing square brackets.

Comment 9

In the Appendix, first sentence of section 3 there are missing parenthesis: Eqs. A17-A19 → Eqs. (A17)- (A19).

Response:

We fixed the missing parenthesis

Comment 10

The caption of Fig. 6 may need improvements. Let us consider the plot relative to DX eff and r1. If I understand correctly, a "trajectory" here refers to a choice of the other 5 parameters (and, possibly, there is more than one trajectory sampled for each combination) for increasing value of r1, from its 1st to its 6th value. Either if this is the case, or something else is instead plotted here, it will be helpful for the readers to have a clearer and more explicit description of the plots.

Response:

We thank the reviewer of this suggestion. We have added the following explanation to the figure captun.

> "Each trajectory correspond to varying the parameter on it's x-axis, while fixing the other 5 parameters"

Comment 11

In the caption of Fig. 6 a period is missing at the end after "Table I".

Response:

The missing period has been added.

Comment 12

References [24] and [30] are the same. (Moreover, typesetting of Ref. [30] is wrong).

Response:

Sorry, we have fixed this mistake.

Authors' Response to Reviewer 3

Comment 1

Energy conservation and the 10% rule: Does the model conserve energy? It seems to me that it does not, in the sense that there is no strict bound on how much biomass can be derived from one trophic level and transferred to the next. This seems a bit strange, since in ecology, people typically think that every subsequent trophic level has 10% the biomass of the previous one, as a result of dissipation and imperfect energy conversion. It would be nice to explore the consequences of such energy loss, since it would make the biomasses of the three trophic levels systematically different. I am curious to see to what extent this would influence the Dtop/Dbottom metrics. In case the authors do not wish to explore this, they could add a note somewhere in the paper.

Response:

This model assumes external energy inflow from environment through carrying capacity k, and dissipation of energy to environment through death of consumers. The classic pyramid biomass distribution (10% biomass of it's resource) can occur for some choices of parameters, reflecting more realistic ecosystems. However, our model also depicts more general ecosystems. For instance, we can imagine a farm where fast growing vegetables are constantly supplied to animals, so total biomass of animal are much larger than vegetables.

To address the reviewers very prescient comments, we have modified our model to include imperfect energy transfer. To do so, we have introduced two two extra parameters, η_X and η_N in range $[0, 1]$ that capture the effect of imperfect conversion. Surprisingly, the D_{top}/D_{bottom} metric is actually unchanged and seems not be effected by such global energy efficiencies. We have noted this in the manuscript

> In model set-up: "and $\eta_X, \eta_N \in [0, 1]$ account for finite efficiency of biomass conversion. When η_X and η_N are close to one, energy is very efficiently transferred across trophic levels. In contrast, when these values are close to zero, energy cannot be efficiently harvested from lower trophic levels."
>
> In discussion of order parameter: "Finally, we note that somewhat surprisingly this order parameter does not *explicitly* depend on the biomass conversion efficiencies η_X and η_N. For this reason, within our model, the effect of imperfect energy conversion manifests itself only through species packing fractions."

Comment 2

Consumption-conversion asymmetry: On a similar note, the authors assume no asymmetry between consumption and growth (biomass conversion), i.e., there is one set of c's and d's. To what extent does that influence the results? It would be nice to add a note about this assumption as well.

Response:

We added trophic level parameters for efficiency of biomass conversion η_N, η_X, which qualitatively does not affect our result. But in general, asymmetry in consumer-resource model or LV model could lead to rich dynamical behavior such as limit cycles and chaos if the level of asymmetry is high enough. We expect similar things happen in this model.

> "While the dynamics of consumer-resource models can display rich behavior, for instance chaos and limit cycles when consumer-resource interactions are asymmetric (non-reciprocal), we choose to focus on the case where the interactions are symmetric. In the physical regime where the mean values of each parameter and the initial biomass of each species is positive, we have found that our numerical simulations always converge to a unique globally stable steady-state for the ecological dynamics. While we currently lack a rigorous proof, we suspect that this reflects the fact that the the multi-trophic consumer resource model possesses a Lyapunov function similar to the two trophic layer system."

Comment 3

Keystone species: Ecosystems can often experience dramatic changes due to the removal or addition of one "keystone" species. I am curious to understand if any of the surviving species in the simulated ecosystems in this paper are of this kind. Specifically, does the theory expect many keystone species or few? Due to the species in a trophic level being statistically similar, it was not clear to me. It seems like in addition to top down and bottom up control, where the carrying capacity or death rate of all species in a trophic level change, one could also compare the scale of these changes with keystone control, where only the presence of one species changes.

Response:

In this work we did not investigate the effect of keystone species. We assumed that all species interactions contribute a order of 1/(number of species) of effect to growth rate. If this assumption

breaks, the calculation in our work will not apply. But it should be possible to include keystone species by reformulating the problem such that very few interaction has a order 1 strength. This is a potentially interesting direction for future work.

Comment 4

Relation between sensitivity and number of levels: The authors show that many of their steady state properties are robust to including more trophic levels. I, however, could not understand if including more levels would suppress the sensitivity to the bottom-up or top-down controls considered here. For instance, would it be the case that whether an intermediate trophic level is controlled by top or bottom depends not just on the species packing fractions of the top or bottom levels, but also on the trophic distance to these levels?

Response:

In this work we defined top-down to bottom-up measurement as a trophic-level-wise quantities. For 3 level models, only herbivores experience both effect from the top and the bottom. While in multiple levels, all intermediate levels experience both effects. While we did not numerically solved cavity equations for more than 3 levels, we added one extra equation to generalize the order parameters to arbitrary levels.

Added equation:

$$\frac{D_{eff}^{i,top}}{D_{eff}^{i,top}+D_{eff}^{i,bottom}} = \frac{\eta^{i+1} r^{i+1} (\sigma_\alpha^{i+1})^2 \chi^{i+1}}{\eta^{i+1} r^{i+1} (\sigma_\alpha^{i+1})^2 \chi^{i+1} + \eta^{i} (\sigma_\alpha^{i-1})^2 \chi^{i-1}}$$

Comment 5

Is it possible to get multiple stable states in the model? The authors state that their model only has a unique stable steady state, but I saw no reference to a proof in the appendix, or a citation to the claim.

Response:

From our simulation we always observe one unique globally stable steady state in our model, except for some extremely non-generic choice of parameters such as when two species have the exact same consumer preferences. While we do not have a formal proof for our specific model

(mostly due to some edge cases we cannot address), we are confident that a unique steady-state is the the generic behavior of a three level trophic system discussed here. We expect that a formal proof can be constructed along the lines of the cited the papers.

In the physical regime where the mean values of each parameter and the initial biomass of each species is positive, we observed that there always exists an unique globally stable steady-state, and a rigorous proof will relies on finding a Lyapunov function similar to those in [33, 34].

Comment 6

It seems by looking at the figures that top-down control is rare — most of the phase diagrams show blue (bottom-up control). Is this just by chance due to the parameters sampled, or is this a broader statement? Even in Fig. 3 the order parameters span three orders on the left of 1, while 2 orders to its right.

Response:

We thank the reviewer for this insightful comment. We realized that the phase diagrams in our original submission could give rise to confusion because of the asymmetric nature of he plotted ratios of the form: D^{top}/D^{bottom}. For this reason, we have changed all plots comparing top-down and bottom-up effects to quantities using fractions. i.e. form of $D^{top}/(D^{top}+D^{bottom})$, which are more intuitive and easier to understand because they treat regions of top-down and bottom-up control more symmetrically.

As the reviewer notes, it is true that bottom-up control dominated regions are more common in our samples than top-down dominated regions. However, it is difficult for us to really make ecologically meaningful statements about real ecosystems since our parameters are sampled uniformly and do not reflect the parameter distributions of real ecosystems. For this reason, we would not read to much into this asymmetry in our figures.

All figures, equation and main text that included $D_{eff}^{N,top}/D_{eff}^{N,bottom}$, $|g_{eff}^{N,top}/g_{eff}^{N,bottom}|$, and $\frac{d\langle N\rangle}{dk}/\frac{d\langle N\rangle}{du}$ are accordingly changed to $D_{eff}^{N,top}/(D_{eff}^{N,top}+D_{eff}^{N,bottom})$, $|g_{eff}^{N,top}|/(|g_{eff}^{N,top}|+|g_{eff}^{N,bottom}|)$, and $\frac{d\langle N\rangle}{dk}/(\frac{d\langle N\rangle}{dk}+\frac{d\langle N\rangle}{du})$

Comment 7

In Fig. 2c and d, I would suggest plotting the yellow curves (middle trophic level) separately from all the others, since that is the one the reader should focus on. Since all the curves are changing, it took me a while to understand that the yellow one was the focus. This could be done by including the other curves in an inset, or just separately.

Response:

We agree that the yellow curves should be stressed.

We added sentences on yellow curves, "Big response" or "Small response", to guide the attention of readers.
